# Direct Observation of Relaxation of Aqueous Shake-Gel Consisting of Silica Nanoparticles and Polyethylene Oxide

**DOI:** 10.3390/polym12051141

**Published:** 2020-05-16

**Authors:** Yi Huang, Motoyoshi Kobayashi

**Affiliations:** 1 Graduate School of Life and Environmental Sciences, University of Tsukuba, 1-1-1, Tennoudai, Tsukuba, Ibaraki 305-8572, Japan; s1830234@s.tsukuba.ac.jp; 2 Faculty of Life and Environmental Sciences, University of Tsukuba, 1-1-1, Tennoudai, Tsukuba, Ibaraki 305-8572, Japan

**Keywords:** shake-gel, bridging effect, direct observation, relaxation time

## Abstract

Controlling the rheological property of suspensions consisting of colloidal particles and polymers is necessary in industry. Especially, gels induced by shear (shake-gel) are interesting phenomena in rheological field. To gain insight into the shake-gel phenomena of the aqueous suspensions of silica nanoparticles and poly(ethylene oxide) (PEO) and its temporal change, we observed the state transition and measured the viscosity of the silica-PEO suspensions. Our results showed that PEO dose, pH, and molecular weight of PEO influence the state of suspension greatly, and revealed the differences of the suspension states, namely, cloudy, permanent gel, shake-gel, and high viscosity sol. We found that the relaxation time from shake-gel to flowable sol increases to the maximum and decreases again with increasing PEO dose. Shake-gels at pH 8.4 relaxed more slowly than at pH 9.4, and shake-gel did not form at pH above 10 in most of cases, indicating high pH inhibits the formation of shake-gels. PEO of molecular weight of 1000 and 4000 kDa easily bonds more silica nanoparticles by bridging and results in the formation of gels with more stable polymer networks. PEO of molecular weight of 1000 and 4000 kDa also led to longer relaxation time of the silica-PEO suspensions from gel to sol.

## 1. Introduction

Aqueous colloidal suspensions containing silica nanoparticles are widely found in geo-environment technological materials, foods, cosmetics, and medicines [1,2]. For such colloidal silica suspensions, it is important to control their rheology. Adding polymers into these colloidal silica suspensions is an effective method [3,4]. The addition of polymers changes the particle-particle interactions and the rheology of silica suspensions. Therefore, it is necessary to reveal the rheology of nano silica suspensions in the presence of polymers.

Some suspensions containing colloidal particles with/without polymers show the change in their viscosity with shear rate [5,6,7,8]. The increase in viscosity with shear is called shear-thickening [7,8]. As a further phenomenon of shear-thickening, some suspensions gel by shaking. Colloidal suspensions showing this phenomenon are called shake-gels [8,9,10]. Cabane et al. [8] showed that colloidal silica suspensions with added poly(ethylene oxide) (PEO) can be shear induced gels. They examined the condition of mixed suspension under several silica and PEO concentrations and reported that the suspensions gel at moderate PEO concentrations. In other words, the suspension cannot form shake-gels at too high or too low PEO concentrations. They proposed that one PEO chain adsorbs to several silica particles and make a necklace-like structure for the first time. Shear stretches the polymers and aligns the necklaces. Then, necklaces are connected to each other to form a gel network. They advocated for a possible mechanism of shake-gels and reported the states of the suspensions at different adsorbed amounts of PEO. Nevertheless, they did not examine the effect of pH; while pH changes the hydrogen bonds, the electrical repulsive force of particles, and thus the bridging effect. 

Zebrowski et al. [9] reported a kind of shake-gel that consists of synthetic clay particles laponite and PEO. They found that shake-gel occurs in a limited range of laponite and PEO concentrations. They proposed that the gelation is because of the bridging of PEO among laponite particles via hydrogen bonding. They measured the time that shake-gels return to sol state (called relaxation time) by direct observation and reported that the relaxation time of the shake-gels decreases with increasing PEO concentration. Furthermore, they found that a small change in PEO concentration can lead to a change up to 10^4^ times in relaxation time. Mar Ramos-Tejada et al. [10] used silica, laponite, and bentonite particles to prepare shake-gels. They examined how the state of the suspension depends on the weight fraction of silica and PEO and plotted the results into a state diagram. They used the PEO dose per silica surface area to evaluate PEO coverage on silica surface. As a result, they revealed that the suspension of silica and PEO has state transitions of no shake-gel, too viscous, shake-gel, and phase separation, with increasing concentrations of PEO. They showed that the complex modulus increases with gelation. As a common problem, however, they did not consider the effect of pH, and few photos recording the gelation and relaxation processes were reported in these studies. It makes it difficult to observe the condition and the temporal change of the gels during the relaxation process. Furthermore, the relaxation time of suspension was not measured. Some other previous studies [11,12,13,14] examined the rheology of shake-gels, but the discussion of the fundamental mechanism of gelation is insufficient and the experimental conditions are limited. This situation leads to difficulties in developing the detailed understanding of shake-gel. 

Most of the previous studies mentioned above pointed out PEO concentration as the factor influencing the state of silica-PEO suspensions. However, pH may also be an important factor that determines the state of suspension. pH affects the adsorption amount of PEO [15], and this may also affect the adsorption state of polymers. Furthermore, pH determines the charge and zeta potential of silica nanoparticles. Zeta potential relates to the electrical repulsion between silica nanoparticles. Electrical repulsion influences the aggregation-dispersion of silica [16,17] and contributes to the determination of state of suspension [8]. Kawasaki et al. [18] examined the effect of pH and revealed that the suspensions change from cloudy sol, permanent gel, shake-gel, and high viscosity sol with increasing pH. They speculated that sufficient repulsion is necessary to keep gel state. Otherwise, the nanoparticles aggregate and the suspension becomes cloudy. However, they did not study the effect of molecular weight of PEO and did not show comprehensive and temporal change in the suspension states. Shi et al. [19] reported that pH affects the conformation of PEO adsorbed on the silica surface, but the conformational transition of PEO in shake-gel range was not discussed.

Moreover, no previous studies provide a reproducible method to shake the suspensions. They used hands to shake the suspensions, or even did not mention the method. This makes it difficult to reproduce the shake-gel phenomena.

In this context, aiming to improve the understanding on the mechanism and rheology of shake-gels, we carried out the measurement of viscosity and examined the state of the silica-PEO suspension by direct observation. We took a series of photos to confirm the detailed temporal change of the suspension during the relaxation process from gel to sol and summarize the relaxation time with different compositions of suspensions. With these results, we observed the states of suspensions carefully, evaluated the rheology and the relaxation process of shake-gel quantitatively, and try to explain the mechanism of shake-gel. Through this study, we figure out the condition to prepare shake-gels and the factors which affect the relaxation process. This information is valuable when we prepare the gel products, which satisfy our needs of rheology and relaxation time, and perform detailed rheological measurements in future. We considered that the change in state diagrams with various conditions is available as a fingerprint of colloidal silica-PEO suspensions.

## 2. Materials and Methods

### 2.1. Materials

Silica nanoparticle suspension (LUDOX TM-50, Sigma-Aldrich, Tokyo, Japan) was used as received without any further purification in this study. The mass fraction of the silica suspension was 49.9%. The hydrodynamic diameter of the particles was 32.35 ± 0.22 nm [18]. Silanol groups on the surface of silica particles deprotonate in water, and thus the silica surface has negative charge. The charge density and zeta potential of silica surface vary with pH and ionic strength [20].

Poly(ethylene oxide) (PEO) is a non-ionic linear polymer and was used to prepare shake-gels in our study. PEOs with average molecular weights of 400, 1000, 4000 kDa (372773-250G, 372781-250G, 189464-250G, respectively) were purchased from Sigma-Aldrich (Tokyo, Japan). Szekely et al. [21] reported that the polymers synthesized by classical polymerization are polydisperse polymers. Without more details from the manufacturer, we consider that the polymers we used are polydisperse. The polydisperse polymers were used in experiments without any further treatments. Stock solutions of PEO were prepared by dissolving PEO powder in deionized water and followed by stirring for 72 h to ensure complete dissolution. The PEO mass fraction of stock solution was set to 1.5%. 

Deionized water (Elix Advantage 5, Millipore, Tokyo, Japan) was used to prepare the solutions and suspensions. The electric conductivity of freshly prepared deionized water was about 0.07 µS/cm. We used HCl and NaOH solutions (Wako Pure Chemical, Fujifilm, Osaka, Japan) to control the pH of the suspensions. All the experiments were conducted at room temperature (20 °C).

### 2.2. The Preparation of Silica-PEO Suspensions

We prepared silica-PEO suspensions in glass test tubes. The inner diameter of the test tubes is 15 mm. The silica suspension and the PEO stock solution were used to prepare the silica-PEO suspensions. The whole mass of the silica-PEO suspension was set to 2 g. The mass fraction of silica was 20% throughout. The added mass of PEO stock solution was changed to vary the dosed mass of PEO per unit silica surface area (called *C*_p_ in this study), to be from 0.02 to 0.15 mg∙m^−2^. We used *C*_p_ instead of the adsorbed amount of PEO, because the adsorbed amount seems to change in the gelation and relaxation process. A proper amount of 1 M HCl or NaOH solution was added to control the pH in a range from 2.5 to 10. The amount of the suspensions and solutions to be added were calculated, and these were added by the order of the silica suspension, deionized pure water, pH adjuster, and the PEO solution. Prepared suspensions were pre-mixed by a test tube mixer (PresentMixer, TAITEC, Nagoya, Japan) for several minutes. Some suspensions gelled in the pre-mixing process. Thus, the suspensions were left stand for about 24 h to ensure the relaxation of these gelled suspensions to sol. 

### 2.3. The Method of Shaking the Suspensions

As mentioned in the introduction, previous studies [10,18] did not mention how the suspension was shaken and thus did not provide a reproducible method to shake these suspensions. Shaking the suspensions by hand may be an effective method to make the suspensions gel. Nevertheless, the amplitude, degree, and frequency of the shaking by hand, which influence the gelation process remarkably, are difficult to be reproduced. To avoid the uncertainty of shaking and to get enough shear rate to make the suspensions gel, we used the same test tube mixer as used in pre-mix to shake the suspensions. We took a video to show how we shook the test tubes (Video S1). The mixer gives rotation horizontally and vertically, and the rotation speed is about 2800 rpm. Suspensions after pre-mixing were left stand for 24 h, and then we pressed the test tubes to the mixer lightly and vertically. Collini et al. [11] showed that a certain minimum time of shaking is necessary to induce gelling. In our preliminary experiments, the minimum time was found to be about 10 s. Therefore, we shook the suspensions for 60 s. Immediately after the shaking process, we left the suspensions to stand for relaxation. Then, we took photos to record the relaxation process until the suspensions relax or up to 24 h. After taking the photos, the pH of the suspensions was measured by a compact pH meter (LAQUAtwin pH-22B, HORIBA, Kyoto, Japan).

### 2.4. The Measurement of Viscosity

The measurement of viscosity was conducted by a rotational cone-plate type viscometer (MerlinVR, Rheosys, Hamilton Township, US). The shear rate was changed from 10 to 5000 s^−1^ by 20 steps, and at each shear rate, the suspensions was sheared for 5 s, and the viscosity was measured for 5 s. After the measurement of viscosity, the pH of the remained suspensions was measured.

## 3. Results and Discussion

### 3.1. State Diagram

**Figure 1:**
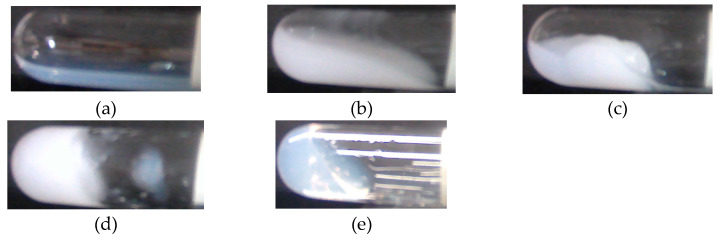
The state of suspension before and after shaking. (**a**) Before shaking, translucent, (**b**) After shaking, cloudy, (**c**) After shaking, shake-gel, (**d**) After shaking, permanent gel, (**e**) After shaking, high viscosity. The pH was 9.4. Poly(ethylene oxide) (PEO) of 1000 kDa was used. The inner diameter of the test tubes is 15 mm.

The silica-PEO suspensions changed their state before and after shaking by the test tube mixer. Representative states of the suspensions are shown in Figure 1. Figure 1a is the suspensions before shaking. It is translucent and behaves as sol. Figure 1b–e are suspensions after shaking. Figure 1b shows a completely turbid sol, which is stated as cloudy. For gelling suspensions, some of the gels turn back to sol state. We call such gels ‘shake-gel’ (Figure 1c). Some gels keep their gel state for a long time, say, more than several months. Such gels are called ‘permanent gel’ (Figure 1d). Furthermore, some suspensions keep translucent and viscous sols after shaking (Figure 1e).

**Figure 2:**
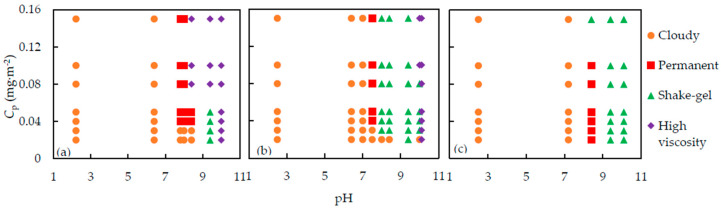
The state diagram of silica (20 wt %) -PEO suspension. The PEO molecular weights are: (**a**) 400 kDa, (**b**) 1000 kDa and (**c**) 4000 kDa. *C*_p_ is the dose of PEO per unit silica surface area.

The results of the state of the suspensions are summarized in Figure 2, in which the symbols stand for different states, cloudy, permanent gel, shake-gel, and high viscosity sol, observed at various pH and *C*_p_. 

From Figure 2, we can find out that pH, *C*_p_ and molecular weight of PEO are the factors which influence the state of silica-PEO suspensions. The shake-gel phenomena occur in a pH range of 8 to 9.4. If pH is lower than 7, the suspensions turn to cloudy sols, meaning the aggregation of the silica nanoparticles. For pH above 10, almost all the suspensions are translucent high viscosity sol, except the suspensions consisting of 4000 kDa PEO, indicating that no silica flocs appeared. *C*_p_ is a parameter to evaluate the dose of PEO per silica surface area. With increasing *C*_p,_ the suspensions changed in the order of cloudy, permanent gel, shake-gel, and high viscosity sol [9,10,14,22]. We also observed a similar tendency around pH 8. However, it seems that the effect of pH is more significant than *C*_p_. Consequently, we found that at low pH, say below pH 7, no matter how *C*_p_ changes, the suspensions are cloudy, and almost all the suspensions at high pH are high viscosity sols. Moreover, we can find that the gelled area, permanent gel and shake-gel, of 4000 kDa PEO suspensions, is the largest. Thus, we consider that the high molecular weight of PEO promotes the formation of gel.

**Figure 3:**
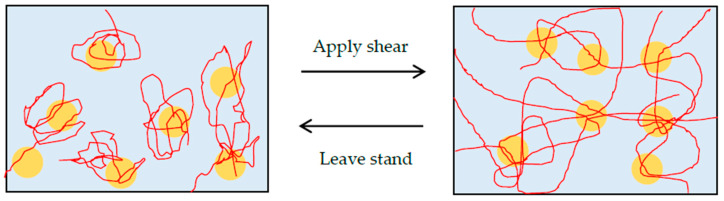
The schematic representation of silica (**circle**) -PEO (**lines**) suspension. Before shaking, the polymers keep random coil state. The suspension is sol. After shaking, the shear flow stretches the polymer chains, and makes bridges between particles. Therefore, the suspension turns to gel state.

As a mechanism of gelation, we consider that the bridging effect through hydrogen bonds between PEO chains and silica particles is the primary prerequisite of shake-gel, as mentioned in previous studies [9,10,11,14,18]. The states change due to shear can be explained by the PEO conformation. Added PEO can be adsorbed to the silanol groups on the silica surface by hydrogen bonds [15]. Even though the enthalpy of displacement of water by PEO is not high [23], so many monomers of PEO can bind to the silica surface. Before shaking, the PEO is adsorbed to the surface of silica, and the conformation remains random-coil-liked. Once a shear flow is applied to the suspension, the PEO is stretched to linear-liked or extended conformation by the shear flow. Stretched PEO polymers can adsorb to other silica particles. Consequently, transient PEO bridges between silica particles and whole network are formed (Figure 3). We discuss the effect of *C*_p_, pH, and molecular weight of PEO to the transient PEO bridges below in more detail.

**Figure 4:**
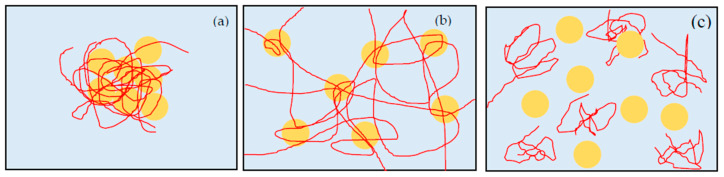
The schematic representation of silica (**circle**) -PEO (**lines**) suspension. The states of these figures are: (**a**) pH lower than 8, (**b**) pH around 9.4, (**c**) pH higher than 10. When pH is low, the electrical repulsion between particles is small. Particles get together to form flocs. When pH is around 9.4, repulsion between particles increases and suspension turns to gel state. If pH increases above 10, the adsorbed amount of PEO decreases steadily, and the suspension keeps the high viscosity sol state.

pH changes the surface charge and zeta potential of silica via the protonation and de-protonation of silanol groups on the silica surface [24,25,26]. The isoelectric point (IEP) of silica is about pH 2. Silica particles have low negative zeta potential around pH 2, and the magnitude of zeta potential increases with increasing pH. At IEP, no electrical repulsion exist between silica particles, and the adsorbed PEO amount is high [15]. Therefore, PEO binds the particles strongly, and the particles get together to make dense flocs (Figure 4a). We can see large whity flocs in this suspension. For suspensions at pH below 7, even though the zeta potential magnitude and electrical repulsion increase with pH, the repulsion is smaller than the bridging attraction, and is not enough to form gel state [18]. When pH increases to about 8, the electrical repulsion increases further. Therefore, silica particles do not form dense flocs but keep an appropriate distance each other. At this condition, PEO polymers make bridges among the particles to create gel network, and the suspension turn to the gel state. However, the adsorption of PEO is still strong and the gel network is too stable to be broken. Therefore, once the suspension turns to gel state, it cannot go back to sol state anymore. At about pH 9.4, the electrical repulsion increases more and the adsorbed amount of PEO decreases. Shear flow stretches the PEO polymers. However, once the shear flow disappears, PEO opts to return to random coil. Then, the gels return to sols because of the desorption of PEO and have a state of shake-gel (Figure 4b). When pH increases above 10, the electrical repulsion is large and PEO adsorbed amount is quite low [11,15]. Hence, the suspension has a state of high viscosity sol, probably due to the increased free PEO concentration (Figure 4c). 

**Figure 5:**
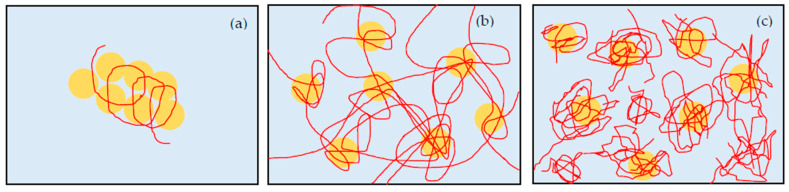
The schematic representation of silica (**circle**) -PEO (**lines**) suspension. The states of these figures are: (**a**) low *C*_p_, (**b**) Appropriate *C*_p_, (**c**) too high *C*_p_. When *C*_p_ is low, the amount of PEO is not enough to form a gel network. The suspension is cloudy sol. When *C*_p_ is appropriate, the number concentration of bridges increases to make bridges among particles. Motion of water is limited in the network, and the suspension turns to gel. If *C*_p_ is too high, available adsorption site of silica decreases. PEO is difficult to form bridges between particles, and suspension keeps high viscosity sol.

*C*_p_ is the dose of PEO polymer and also determines the state of suspensions. When we apply the shear flow to silica-PEO suspension, absorbed PEO is stretched and binds other silica particles by bridging effect. However, when *C*_p_ is low, the amount of PEO chains is not enough to form a network in whole suspension to fix water molecules. Therefore, the suspension turns to the cloudy state after shaking (Figure 5a). With the increase of the PEO, the number concentration of polymer bridges increases. PEO bridges make a strong network among the flocs, and the motion of water is limited in the network. Suspensions in this condition show a state of gel (Figure 5b). If the PEO concentration increases further, the adsorbed amount of PEO increases and the adsorption sites of silica surface are occupied by PEO polymers. It means that the available adsorption site for bridging polymers on silica surface decreases, and PEO chains are difficult to make bridges between the particles. Therefore, neither flocs of particles nor the gel network is formed, and thus the suspension remains as high viscosity sol state (Figure 5c). The viscosity is high because of the high mass fraction of PEO in the bulk [27]. However, the effect of *C*_p_ seems to be influenced by pH, as if pH is low, the suspension trends to get cloudy instead of gel or high viscosity sol, though *C*_p_ is high. If pH is very high, almost all the suspensions tend to be high viscosity sol, regardless of *C*_p_. We consider that it is because pH influences the adsorbed amount of PEO. At low pH, even though the dose is high, almost the PEO adsorbed to silica surface strongly and form flocs, because the dose is smaller than the saturation [15]. However, at very high pH, the adsorbed amount is low, regardless of *C*_p_. Therefore, the suspensions have a trend to be high viscosity sol.

The molecular weight of PEO relates to the number of monomers in one polymer, hereby influences the state of suspensions. At determined condition of pH and *C*_p_, the state of suspension changes from cloudy to gel, and then to high viscosity sol. For 400 kDa PEO, the number of monomers in a PEO polymer is low. Therefore, the number of hydrogen bonds between PEO and silica per polymer chain is low. The radius of gyration *R*_g_ is 35.9 nm, which is almost the same as the size of the silica particle [18,28]. This makes one polymer able to bind only a few particles. Furthermore, short polymers are difficult to form network among the whole suspension, so the particles trend to aggregate to flocs, and the suspensions become cloudy. For 1000 and 4000 kDa PEO polymers, the *R*_g_ is about 61.0 nm and 136.3 nm, respectively, and larger than silica particles size, so that one PEO can bind more particles. The number of hydrogen bonds per PEO chain is also high. Therefore, high molecular weight polymers promote the formation of gels and inhibit the relaxation of gels. Nevertheless, we should keep in mind the effect of pH. Too low pH may make the bridging effect too strong and induces cloudy suspensions. As a result, at most cases around pH 7.2, the suspensions with 1000 kDa PEO form permanent gels, but the suspensions with 4000 kDa PEO form cloudy sols.

### 3.2. Photos and the Relaxation Time of Suspensions

We took photos of the suspensions from immediately after the shaking to 24 h to confirm the temporal change of state of suspensions (Figures S1–S5). The state of the suspensions can be roughly classified into sol and gel. In this paper, we focus on the direct observation of the gelled ones. 

**Figure 6:**
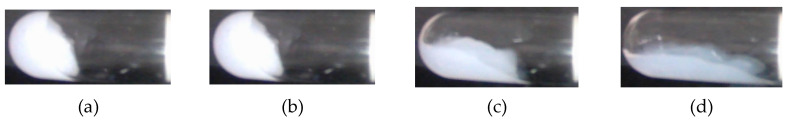
The state of suspension after shaking. (**a**) Gel, making a large angle or even be vertical to the ground, (**b**) Keeping sticking to the upper wall of test tubes, (**c**) Slid or flowed down from the upper wall, (**d**) The angle between interface and ground decreases continuously. The inner diameter of the test tubes is 15 mm.

**Figure 7:**
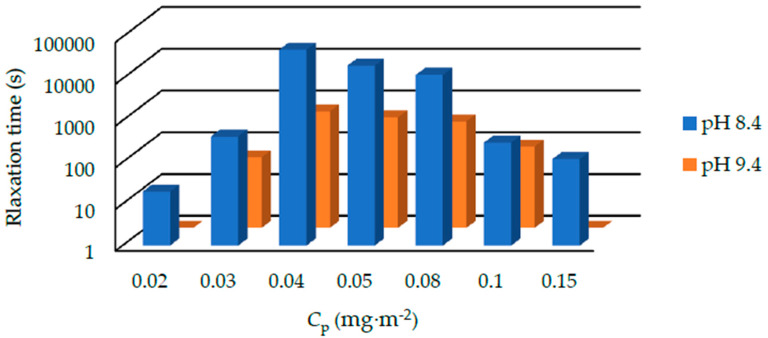
The relaxation time of silica-PEO suspensions consist of 1000 kDa PEO under the pH of 8.4 and 9.4. The temperature is 20 °C. For suspensions of pH 10, almost all the suspensions do not form gel state. We cannot measure the relaxation time of them.

**Figure 8:**
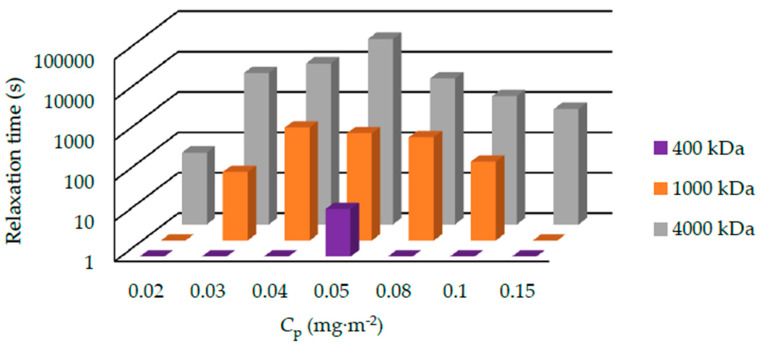
The relaxation time of suspensions consist of 400, 1000, and 4000 kDa PEO under the pH of 9.4. The temperature is 20 °C. The suspensions which consist of 400 kDa PEO slid or flowed down so quickly that we cannot measure the relaxation time of almost all of the suspensions.

In the relaxation process, we can find some obvious changes in the states of suspensions. Immediately after the shaking, the suspensions show a state of gel. For gelled suspensions, the elasticity of gel can support their weight themselves, so the interface between gel and air can make a large angle or even be vertical to the ground (Figure 6a). As time passes, the gels begin to relax, and the angle between interface and ground decreases. In this regime, the gels keep sticking to the upper wall of test tube (Figure 6b). Then, the elasticity of gel decreases again in time, and the silica-PEO mixture becomes no longer able to support the weight. Therefore, gels slide or flow down from the upper wall of test tubes (Figure 6c). After this regime, the angle between interface and ground decreases continuously and finally becomes horizontal (Figure 6d). Measuring the relaxation time of the suspensions can help us to understand the effect of pH and *C*_p_. However, it is difficult to measure the time when the interface becomes horizontal correctly. Thus, in this study, we define the condition that the gel sticks to the half of the bottom of test tube as a relaxed condition (Figure 6c). The time from finishing the shaking to the relaxed condition is defined as a relaxation time. For suspensions relaxing in 1 h, the time interval of photos is 3 s. For suspensions whose relaxation time is more than 1 h, the time interval of photo is 30 s. We obtained the relaxation time from these photos and summarized the results in Figure 7 and Figure 8.

The relaxation time of suspensions with 1000 kDa PEO under the pH of 8.4 and 9.4 is shown in Figure 7. The relaxation time varies from several minutes to several hours. Generally, the relaxation time at pH 9.4 is shorter than that at pH 8.4. We also summarized the results of suspensions containing 400, 1000, and 4000 kDa PEOs at pH 9.4 in Figure 8. For 400 kDa silica-PEO suspensions, we see all the relaxation times are several seconds. It is too short to measure the relaxation time of most of the suspensions correctly. The relaxation time of suspensions with 1000 kDa PEO is longer. For 4000 kDa suspensions, the relaxation time is very long. In both the conditions of pH 8.4 and 9.4, and all the condition of suspensions with 400, 1000, and 4000 kDa PEOs, we can observe that the relaxation time increases to the maximum at first and then decreases with *C*_p_. This trend cannot be noted just from the state diagram.

As for a reason of the effect of pH on the relaxation time of shake-gel, we consider that this is due to the change in the adsorbed amount of PEO and electrical repulsion between silica nanoparticles. When pH is low, the adsorbed amount of PEO is high [15] and the electrical repulsion between silica nanoparticles is low [24]. Therefore, the bonds of PEO and silica are difficult to break up, and silica nanoparticles are hard to be dispersed. Consequently, the relaxation time at pH 8.4 is longer than that at pH 9.4.

Under the same pH and *C*_p_, we can see that the relaxation time of silica-PEO suspensions increases with the molecular weight of PEO. PEO with larger molecular weight has long loop and tail of adsorbed chains. Long loop and tails mean that the polymer can attach silica at more points and trap more particles, and a stronger network is formed. Therefore, the binding between PEO and silica nanoparticles and the gel network are more stable. Additionally, stretched chains with higher molecular weight under exerted force are calculated to relax slowly to equilibrium after the force is released [29]. Adam et al. [30] reported that large molecular weight polymers have a long rheological relaxation time from their experiments. These mean that large polymers need more time to return to random coil state. For these reasons, the suspensions with higher molecular weight PEO have a long relaxation time. On the contrary, the PEOs with molecular weight of 400 kDa have a shorter loop of monomers. Thus, the gel is difficult to form and the relaxation time is shorter. 

**Figure 9:**
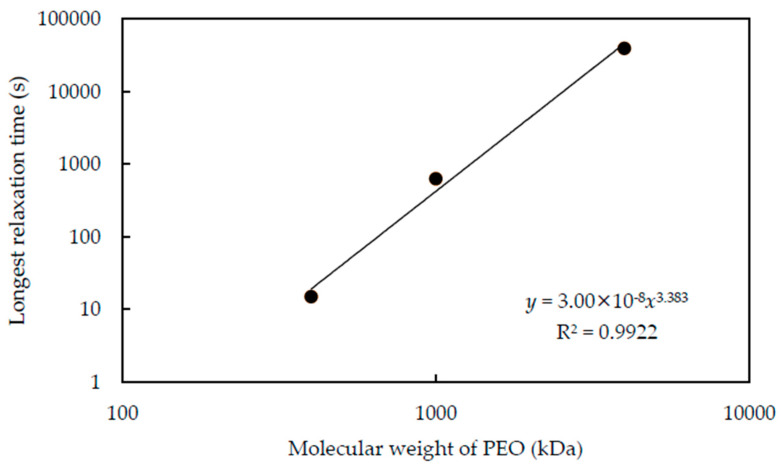
The longest relaxation time of suspensions consist of each molecular weight PEO under the pH of 9.4. The temperature is 20 °C. The longest relaxation time increases with the increasing molecular weight of PEO, and the relaxation time seems to have a relationship of power function with molecular weight.

While the relaxation time changes with the pH and *C*_p_, it is interesting to discuss the relationship between longest relaxation time and molecular weight. We summarize the longest relaxation time of each molecular weight PEO at pH 9.4 in Figure 9. We can confirm that the longest relaxation time increases with the increasing molecular weight of PEO and follows the power-law relationship. The power is 3.38, which is almost the same as the power for the relationship between molecular weight and the relaxation time of polymeric fluids [31]. This means that the polymers are constrained by entanglement [31] as well as adsorption.

*C*_p_ also influences the adsorbed amount and thus changes the amount of available adsorption site of silica surface. When *C*_p_ is low, increasing PEO dose promotes the bridges between particles. The gel structure gets more stable, and the relaxation time increases and reaches the maximum. If *C*_p_ increases further, the available adsorption sites on the silica surface decrease. This makes it difficult for each polymer to form bridges among many particles. In this condition, PEO polymers are easier to desorb from particle surface, because of fewer hydrogen bonds. Thus, the relaxation time decreases. Furthermore, we find that the longest relaxation time occurs at around *C*_p_ = 0.04–0.05 mg∙m^−2^ irrespective of pH and the molecular weight of PEO. This is such an interesting result, that PEO dose per surface area seems to be the most important factor to determine the relaxation of shake-gel. This also supports the hypothesis that a sufficient amount of the available adsorption site is necessary to build and keep the bridges between particles. Again, this discussion is now possible from our novel observation on the relaxation and was impossible from only the state diagram. 

Moreover, we consider the measurement of viscoelasticity as a better method to evaluate the gelation and relaxation. We will conduct some experiments about viscoelasticity in future research. Before such measurements, our results are convenient as a fingerprint of the states.

The photos used to confirm the states and calculate the relaxation time are summarized in the Supporting Information (Figures S1–S5).

### 3.3. The Results of Viscosity Measurement

**Figure 10:**
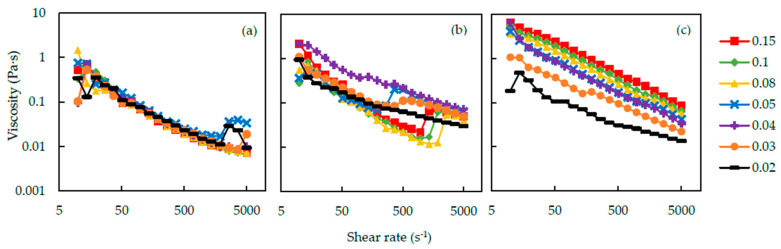
The viscosity of silica-PEO suspensions against shear rate. The symbols represent the *C*_p_(mg∙m^−2^). The conditions of these suspensions are (**a**) 400 kDa, pH 9.4, (**b**) 1000 kDa, pH 9.4 and (**c**) 1000 kDa, pH 10.

Figure 10 shows the relation between viscosity of the silica-PEO suspensions and shear rate. The conditions of these suspensions are (a) 400 kDa and pH 9.4, (b) 1000 kDa and pH 9.4 and (c) 1000 kDa and pH 10. In all of the figures, the viscosity decreases with the increasing shear rate at first. This phenomenon is called shear-thinning [31]. Nevertheless, particular suspensions show an increase of viscosity in the process of increasing shear rate. We call this phenomenon shear-thickening. Because the viscosity increase occurs in a gelation process, we consider the shear-thickening phenomenon as a symbol of gelation induced by shear flow. Under the condition of PEO molecular weight 1000 kDa and pH 9.4 in Figure 10(b), the suspensions seem to show the shear-thickening phenomenon. At first, the viscosity of the suspensions decreases with the increase of shear rate. At a critical shear rate, however, the viscosity of suspensions increases sharply, and at the same time, the gelation of the suspensions was observed. When the shear rate reaches the critical shear rate, it also takes some time for the suspensions to form gel [11]. When the PEO concentration is not too high, shear flow stretches the PEO chains to adsorb to multiple particles, because of the large amount of bare silanol group on the surface of silica particle (Figure 5b). Polymer bridges connect and trap the particles and increase the size of particles’ clusters. Therefore, the viscosity increases. After this increase in viscosity, high shear rate breaks the clusters and the viscosity decreases again. With increasing the concentration of PEO, more PEO polymers are adsorbed to the surface of silica. This induces the decrease of the available adsorption sites of silica. Therefore, PEO chains are difficult to bridge between silica particles, and a higher shear rate is needed to form a gel network (Figure 5c). 

If we look into the result with the PEO molecular weight to 400 kDa, we can observe obvious differences in the viscosity against the shear rate results (Figure 10a). The suspensions with 400 kDa PEO have lower viscosities, and higher shear rate is needed to cause the shear-thickening. The chain length and *R*_g_ of the 400 kDa polymer are smaller [28]. Smaller polymers can only bind fewer particles. Particle clusters become smaller, and thus, the viscosity is lower than that of 1000 kDa silica-PEO suspensions. The viscosity of the suspension with 4000 kDa PEO could not be measured, because the gel was easily formed and could not be set up on the viscometer.

For the suspension with 1000 kDa PEO, when the pH of the suspension increased to 10, almost no shear-thickening phenomenon happened (Figure 10c). At high pH, the adsorbed amount of PEO on silica decrease rapidly [15] and PEO bridges over silica particles are difficult to form. Therefore, the shear-thickening did not occur, and no shake-gel phenomenon was observed at pH 10. The viscosity of suspensions increases with *C*_p_. Furthermore, compared to the viscosity at pH 9.4, the viscosity at pH 10 is higher overall. A previous study [19] reported similar results and advocated that longer distance between bridged silica nanoparticles due to the strong electrical repulsion at high pH is the reason of this phenomenon. We also think that high viscosity at high pH is induced by the decrease in the adsorbed amount of PEO and a large amount of PEO polymers remained in the medium. 

We also find that through the change of *C*_p_, the suspension at *C*_p_ = 0.04 mg∙m^−2^ has the highest viscosity among all of the shake-gelled suspensions. This agrees with the longest relaxation time in Section 3.2 and may indicate that this is the best *C*_p_ condition for stable gel. Furthermore, the suspensions gel in the viscosity measurement process, mainly after the increase of viscosity. Therefore, because of the slipping of gels on the plate of the viscometer, the viscosity is possible to be incorrect after the sudden increase of viscosity. We plan to measure the dynamic viscoelasticity of the gels in future studies to solve this problem.

## 4. Conclusions

To obtain better insight into shake-gels that consist of silica nanoparticles and polyethylene oxide (PEO), we observed the state transition of the suspension, took photos of them to record the process of relaxation, and measured the suspensions viscosity. We confirmed that *C*_p_, pH, and molecular weight of PEO influence the state of the silica-PEO suspension, namely, cloudy, permanent gel, shake-gel, and high viscosity sol. The pronounced shake-gel can be obtained at pH around 9.4 and *C*_p_ around 0.04 mg∙m^−2^. The PEO of molecular weight of 1000 and 4000 kDa can lead to easier formation of shake-gel and longer relaxation time. We confirmed that the longest relaxation time increases with the increasing molecular weight of PEO and follows power-law relationship with a power of 3.38. These findings are useful to prepare the shake-gels with our needs, such as relaxation time and viscoelasticity under a certain range of solution conditions etc. and for further analyses with rheological and scattering instruments. We expect this research to be useful in the development of paint and cosmetics.

## Supplementary Materials

The following are available online at https://www.mdpi.com/2073-4360/12/5/1141/s1, Figure S1: Temporal change of the 20% silica suspensions with different load of PEO with 1000 kDa at pH 9.4. Almost the suspensions are shake-gels. At about *C*_p_ = 0.04 mg∙m^−2^, the longest time is needed to relax. Figure S2: Temporal change of the 20% silica suspensions with different load of 1000 kDa at pH 8.4. Almost the suspensions are shake-gels. However, the relaxation time is longer than that of pH 9.4. At about *C*_p_ = 0.04 mg∙m^−2^, the longest time is needed to relax. Figure S3: Temporal change of the 20% silica suspensions with different load of PEO with 1000 kDa at pH 10. Almost the suspensions are high viscosity sols. Figure S4: Temporal change of the 20% silica suspensions with different load of PEO with 400 kDa at pH 9.4. Almost the suspensions are shake-gels. However, the relaxation time is too short, and they relax before the capture equipment are set. We are not able to measure the relaxation time correctly. Figure S5: Temporal change of the 20% silica suspensions with different load of PEO with 4000 kDa at pH 9.4. Almost the suspensions are shake-gels. The relaxation time is longer than that of 1000 kDa. Video S1: The method to shaking the silica-PEO suspensions. The suspension springed highly at first and acted as a sol. After several seconds, the suspension became gel, and stuck to the bottom of test tube.
