# Supplementary material for: Direct Observation of Relaxation of Aqueous Shake-Gel Consisting of Silica Nanoparticles and Polyethylene Oxide
Source: Polymers (Basel). 2020 May 16;12(5):1141. doi: 10.3390/polym12051141 (PMC7285087; doi:10.3390/polym12051141)
Supplement: Supplementary file 1 [file polymers-12-01141-s001.zip › 20200510 supporting information .docx]

| Time (min)  *C*_p_ (mg∙m^-2^) | 0 | 0.5 | 1 | 5 | 30 | 720 |
| --- | --- | --- | --- | --- | --- | --- |
| 0.02 | 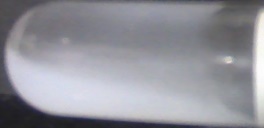 | 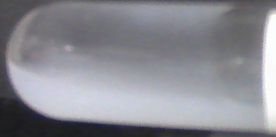 |  |  |  |  |
| 0.03 | 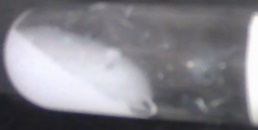 | 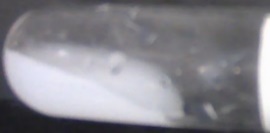 | 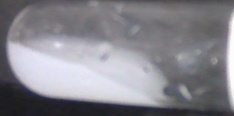 | 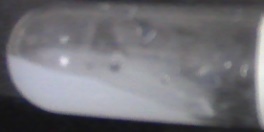 | 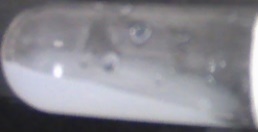 |  |
| 0.04 | 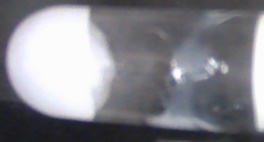 | 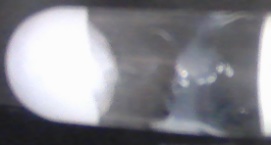 | 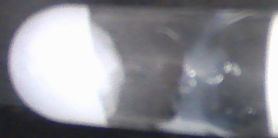 | 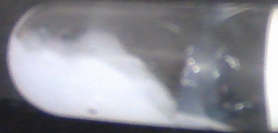 | 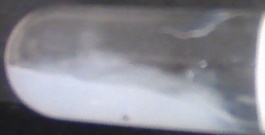 | 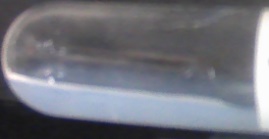 |
| 0.05 | 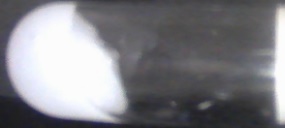 | 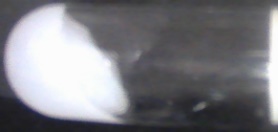 | 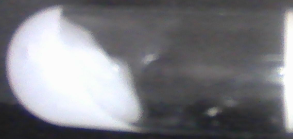 | 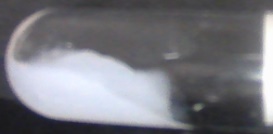 | 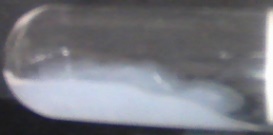 |  |
| 0.08 | 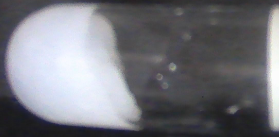 | 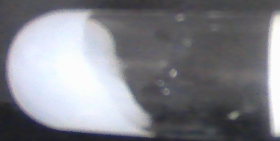 | 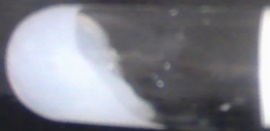 | 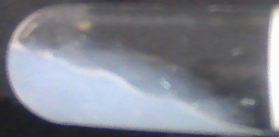 | 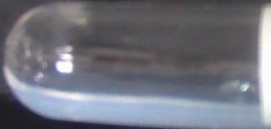 |  |
| 0.1 | 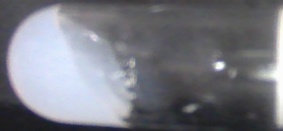 | 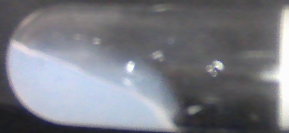 | 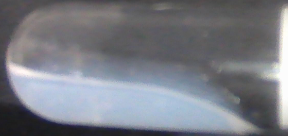 | 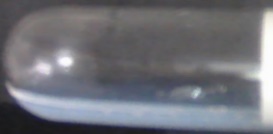 |  |  |
| 0.15 | 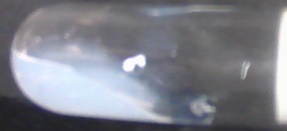 | 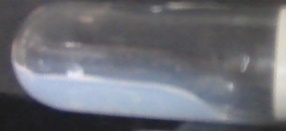 | 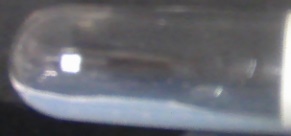 |  |  |  |

Figure S1 Temporal change of the 20% silica suspensions with different load of PEO with 1000 kDa at pH 9.4. Almost all the suspensions were shake-gels. At about *C*_p_=0.04 mg∙m^-2^, the longest time was needed to relax. The inner diameter of the test tubes is 15 mm.

| Time (min)  *C*_p_ (mg∙m^-2^) | 0 | 0.5 | 1 | 5 | 30 | 720 |
| --- | --- | --- | --- | --- | --- | --- |
| 0.02 | 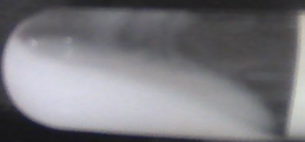 | 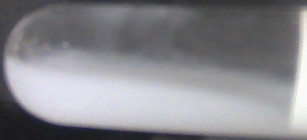 |  |  |  |  |
| 0.03 | 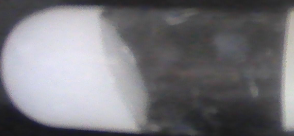 | 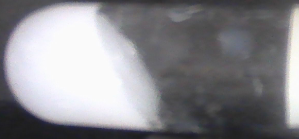 | 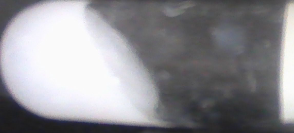 | 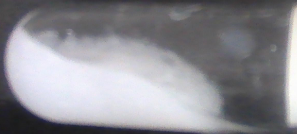 | 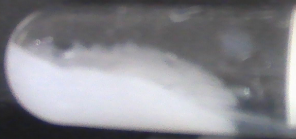 |  |
| 0.04 | 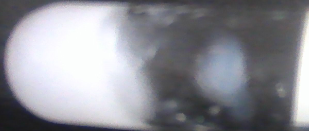 | 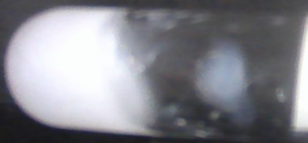 | 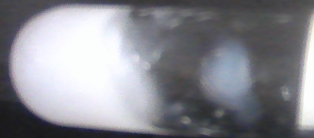 | 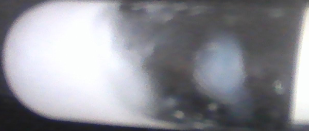 | 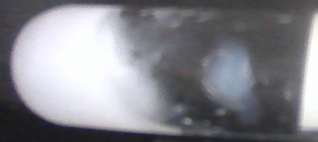 | 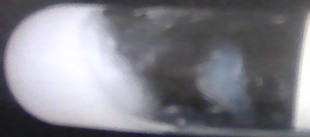 |
| 0.05 | 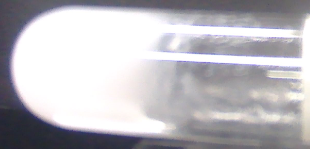 | 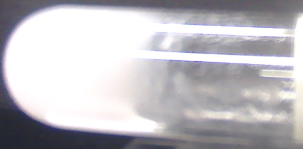 | 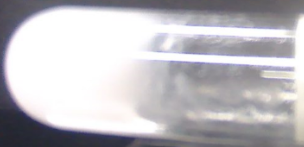 | 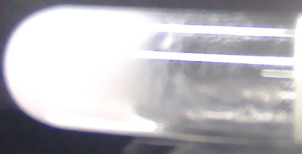 | 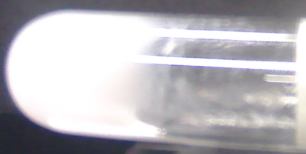 | 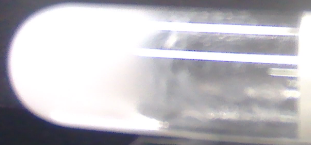 |
| 0.08 | 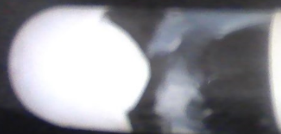 | 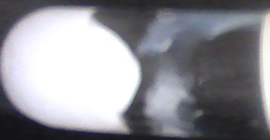 | 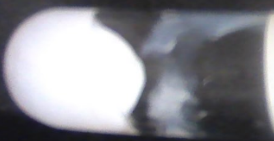 | 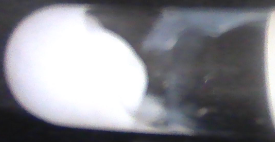 | 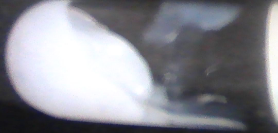 | 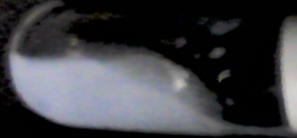 |
| 0.1 | 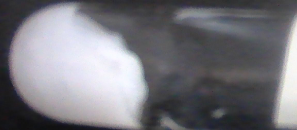 | 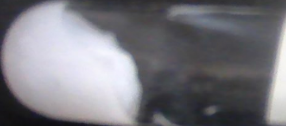 | 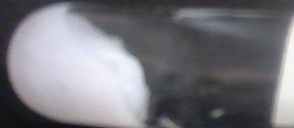 | 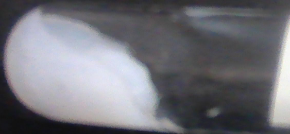 | 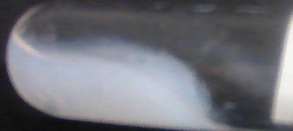 |  |
| 0.15 | 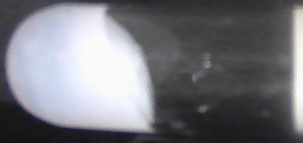 | 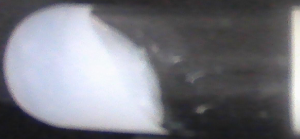 | 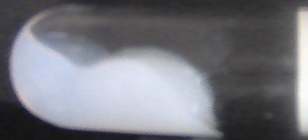 | 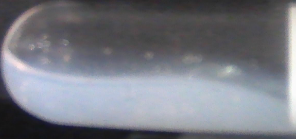 |  |  |

Figure S2 Temporal change of the 20% silica suspensions with different load of 1000 kDa at pH 8.4. Almost all the suspensions are shake-gels. However, the relaxation time was longer than that of pH 9.4. At about *C*_p_=0.04 mg∙m^-2^, the longest time was needed to relax. The inner diameter of the test tubes is 15 mm.

Figure S3 Temporal change of the 20% silica suspensions with different load of PEO with 1000 kDa at pH 10. Almost all the suspensions were high viscosity sols. The inner diameter of the test tubes is 15 mm.

| Time (min)  *C*_p_ (mg∙m^-2^) | 0 | 0.5 | 1 | 5 | 30 | 720 |
| --- | --- | --- | --- | --- | --- | --- |
| 0.02 | 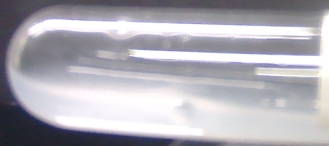 | 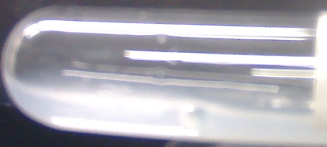 |  |  |  |  |
| 0.03 | 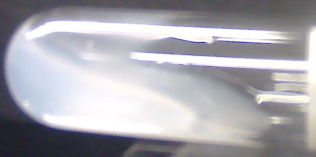 | 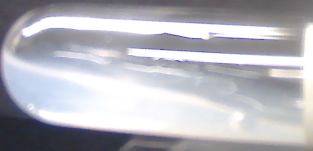 | 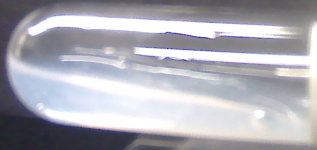 | 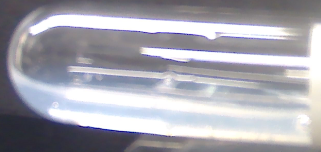 |  |  |
| 0.04 | 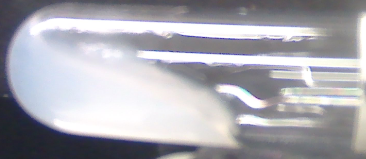 | 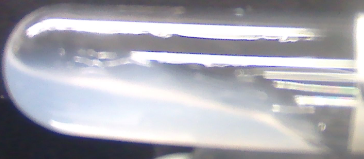 | 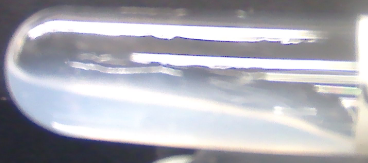 | 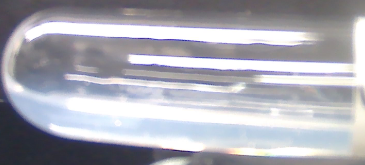 |  |  |
| 0.05 | 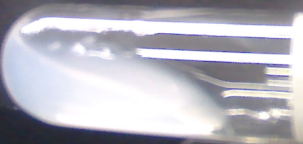 | 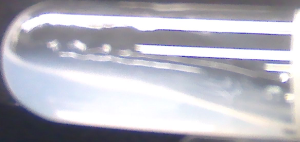 | 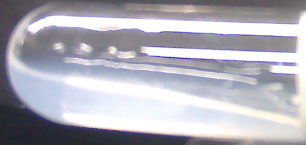 | 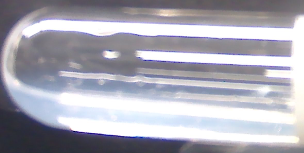 |  |  |
| 0.08 | 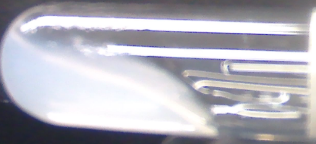 | 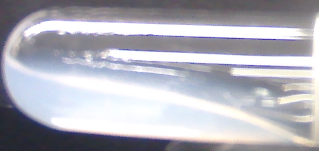 | 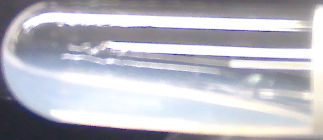 | 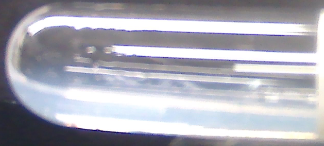 |  |  |
| 0.1 | 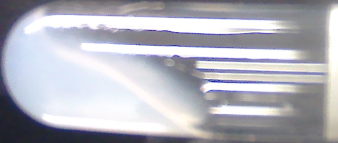 | 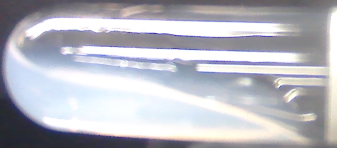 | 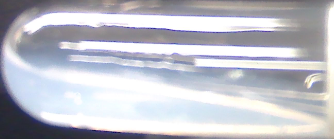 | 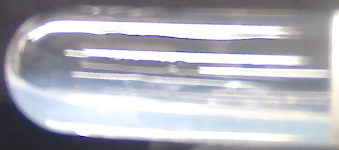 |  |  |
| 0.15 | 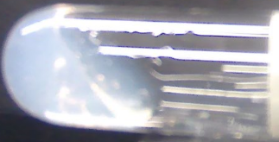 | 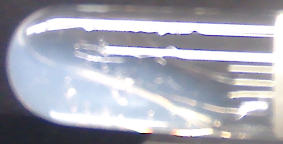 | 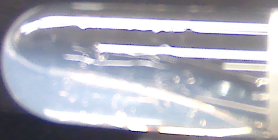 | 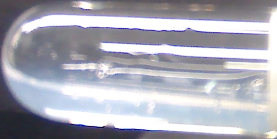 |  |  |

Figure S4 Temporal change of the 20% silica suspensions with different load of PEO with 400 kDa at pH 9.4. Almost all the suspensions were shake-gels. However, the relaxation time was too short, and they relaxed before the capture equipment was set. We were not able to measure the relaxation time correctly. The inner diameter of the test tubes is 15 mm.

| Time (min)  *C*_p_ (mg∙m^-2^) | 0 | 0.5 | 1 | 5 | 30 | 720 |
| --- | --- | --- | --- | --- | --- | --- |
| 0.02 | 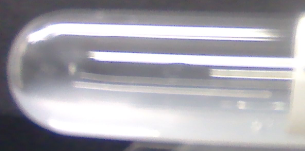 | 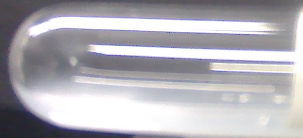 |  |  |  |  |
| 0.03 | 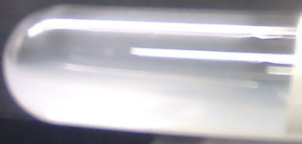 | 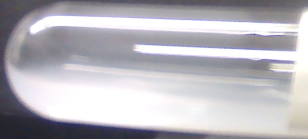 | 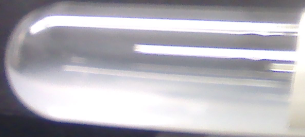 |  |  |  |
| 0.04 | 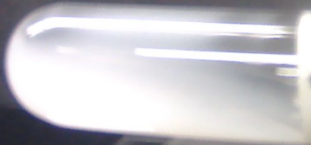 | 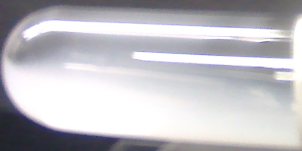 | 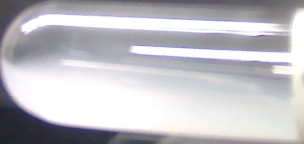 |  |  |  |
| 0.05 | 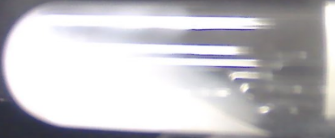 | 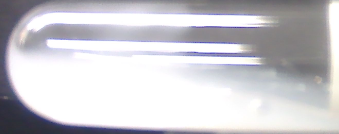 |  |  |  |  |
| 0.08 |  |  |  |  |  |  |
| 0.1 |  |  |  |  |  |  |
| 0.15 |  |  |  |  |  |  |

Figure S5 Temporal change of the 20% silica suspensions with different load of PEO with 4000 kDa at pH 9.4. Almost all the suspensions are shake-gels. The relaxation time were longer than that of 1000 kDa. The inner diameter of the test tubes is 15 mm.

| Time (min)  *C*_p_ (mg∙m^-2^) | 0 | 0.5 | 1 | 5 | 30 | 720 |
| --- | --- | --- | --- | --- | --- | --- |
| 0.02 |  |  |  |  |  |  |
| 0.03 |  |  |  |  |  |  |
| 0.04 |  |  |  |  |  |  |
| 0.05 |  |  |  |  |  |  |
| 0.08 |  |  |  |  |  |  |
| 0.1 |  |  |  |  |  |  |
| 0.15 |  |  |  |  |  |  |
